# iMuseumA: An Agent-Based Context-Aware Intelligent Museum System

**DOI:** 10.3390/s141121213

**Published:** 2014-11-10

**Authors:** Inmaculada Ayala, Mercedes Amor, Mónica Pinto, Lidia Fuentes, Nadia Gámez

**Affiliations:** Departamento de Lenguajes y Ciencias de la Computación, Andalucia Tech, Universidad de Málaga, Campus de Teatinos s/n, 29071 Málaga, Spain; E-Mails: pinilla@lcc.uma.es (M.A.); pinto@lcc.uma.es (M.P.); lff@lcc.uma.es (L.F.); nadia@lcc.uma.es (N.G.)

**Keywords:** ambient intelligence, agent technology, group-based communication, intelligent museums

## Abstract

Currently, museums provide their visitors with interactive tour guide applications that can be installed in mobile devices and provide timely tailor-made multimedia information about exhibits on display. In this paper, we argue that mobile devices not only could provide help to visitors, but also to museum staff. Our goal is to integrate, within the same system, multimedia tour guides with the management facilities required by museums. In this paper, we present iMuseumA (intelligent museum with agents), a mobile-based solution to customize visits and perform context-aware management tasks. iMuseumA follows an agent-based approach, which makes it possible to interact easily with the museum environment and make decisions based on its current status. This system is currently deployed in the Museum of Informatics at the Informatics School of the University of Málaga, and its main contributions are: (i) a mobile application that provides management facilities to museum staff by means of sensing and processing environmental data; (ii) providing an integrated solution for visitors, tour guides and museum staff that allows coordination and communication enrichment among different groups of users; (iii) using and benefiting from group communication for heterogeneous groups of users that can be created on demand.

## 1. Introduction

In the digital era, any visitor to a museum demands advanced services adapted to the different users' profiles. Therefore, over the last few years, most museums have provided handheld devices with vocal instructions, to users, which has introduced a new concept to museum visits: the audio tour. However, these handheld devices or audio guides only offer audio information and have a cost that normally increases the entrance fee. With the popularization of mobile devices, these audio guides are becoming a thing of the past. Instead of using audio guides, museums are currently providing interactive tour guide applications that can be installed in mobile devices [1–7].

Unlike the audio guides, interactive tour guide services provide timely tailor-made multimedia information about the exhibits on display. This means that the information is now presented to the user in a variety of media forms on a portable screen-based device, a tablet or a mobile phone. Most of the interactive tour guide applications available make each visit a personalized experience by tailoring the route and the information displayed according to a user's preferences and the desired length of visit [8,9]. Others provide group communication with the aim of providing better support to professional tourist guides [10].

In this paper, we argue that mobile devices not only could provide solutions to visitors, but also to museum staff. The museum staff have to be able to interact with all visitors, so as to carry out important management activities, for example notifying everybody that the museum is closing soon. Therefore, our goal is to integrate, within the same system, multimedia tour guides with the management facilities required by museums. Some of the management tasks must be supported by sensors, e.g., detecting when a room is empty. With the latest advances in sensor technology, sensors are currently considered to be a technology at anyone's fingertips. They are becoming more powerful, cheaper and smaller in size. Furthermore, including sensors in new generations of mobile devices is a current trend. Therefore, mobile phones, already equipped with multiple wireless interfaces (IEEE 802.11, Bluetooth and 3G), now include onboard sensing solutions together with location capabilities. Moreover, low-power connectivity is expected to become available in the near future, on most consumer devices used in museums. In these situations, it is possible that mobile devices will be able to collect and analyze sensor data from different sources, becoming a usual device for monitoring environmental conditions. This recent technological progress provides a unique chance to use mobile devices to support any kind of activity inside a museum.

In this paper, we present iMuseumA (intelligent museum with agents), a mobile-based solution to provide personalized visits and perform context-aware management tasks. The work presented here takes advantage of new technologies (wireless sensor networks, powered mobile personal devices and an enhanced wireless communication infrastructure) to provide context-aware services for visitors, guides and museum staff in an integrated way. Context-awareness [11] plays a critical role in deciding what data needs to be processed and how to react to the different situations that might arise inside the museum [12]. The data is collected from the environment, but can be analyzed, interpreted and understood in different ways. Furthermore, in order to carry out a coordinated management of tourist visits, iMuseumA provides group communication facilities between the different groups of users (*i.e.*, visitors, professional tourist guides and museum staff). iMuseumA follows an agent-based approach, which makes natural interaction with the museum environment possible, so that decisions can be made based on its current status. Multiagent systems are especially good at modeling real-world and context-aware systems, where problems can be solved in a concurrent and cooperative way [13]. Compared with other similar solutions, the main contributions of iMuseumA are: (i) a mobile application that provides management facilities to the personnel of the museum by means of sensing and processing environmental data; (ii) providing an integrated solution for visitors, tourist guides and museum staff that enables coordination and communication enrichment between different groups of users; (iii) using and benefiting from group communication for heterogeneous groups of users that can be created on demand. Although there are some museum systems that provide automated monitoring, none of them provide this functionality integrated with other apps. Finally, the use and benefit of group communication for heterogeneous groups of users that can be created on demand is something that is not commonly supported by similar museum systems. The iMuse Mobile Tour [10] is one example of this, but it only exploits the group's creation to improve the user's visit and does not support the security staff's and guide's work, nor does it improve the interaction between security staff, visitors and guides.

The iMuseumA system is currently installed and deployed in the Museum of Informatics at the Informatics School of the University of Málaga (Escuela Técnica Superior de Ingeniería Informática in Spanish). In this paper, we principally discuss three mobile applications (commonly known as apps) that are part of the iMuseumA system and are specific to each role identified in the Museum of Informatics. The first app is an enhanced mobile guide for visitors that offers different tours (both guided and without a guide) of the museum rooms. For visitors, the system provides valuable and tailored data according to their location and interests. Visitors' mobile devices normally have the capabilities to detect the user context and to provide relevant pieces of information to help visitors during their visit. The second app is specifically for museum guides, with the aim of helping them to communicate with their groups, to coordinate the routes, exchange tailored information and interact with the museum staff. Additionally, the third app is aimed to assist museum staff in controlling and monitoring the museum's rooms, providing environmental information from sensors and about the current status of the visitors (their location, if they are part of a guided tour group, *etc.*). These applications have been thoroughly evaluated in order to validate the overall performance of their functionalities from the user perspective and effective capability to support the needs of different users in the museum context.

The rest of the paper is organized as follows: Section 2 offers a description of the peculiarities of the museum where our system is installed and deployed and also affords a brief description of the composition of the system, which has been designed as a multi-agent system (MAS). Section 3 provides the necessary background to the agent framework and the agent infrastructure that support the execution and communication of the software agents in the museum system. Section 4 addresses the internal design of agents in sensors, while Sections 5 and 6 describe, respectively, the mobile applications that provide support for users during the visit and those that monitor our museum. Section 7 presents and discusses the evaluation of these applications from the user's perspective. Finally, Section 8 provides a review of existing work on museum guides before Section 9 presents the most relevant conclusions of our work and sketches out future work on our system.

## 2. System Specification: Describing the Museum

The museum at the Informatics School of the University of Málaga was created in 2003 as an initiative to exhibit, in public areas of the building, artifacts and other obsolete equipment with a historical interest for current generations of computer science students. The purpose of this museum is to draw attention to the constant evolution of software and hardware technologies in computer science, showing interesting items and machines that have shaped the beginning of a new information and digital era. The collection comprises several computing artifacts and also includes several posters and panels that explore the evolution of informatics and computer science and their impact on society. The museum also provides the opportunity for students of local secondary schools to take tours of the museum following a regular or a special tour for students. These tours are guided by volunteer students of our own School of Informatics.

**Figure 1.**
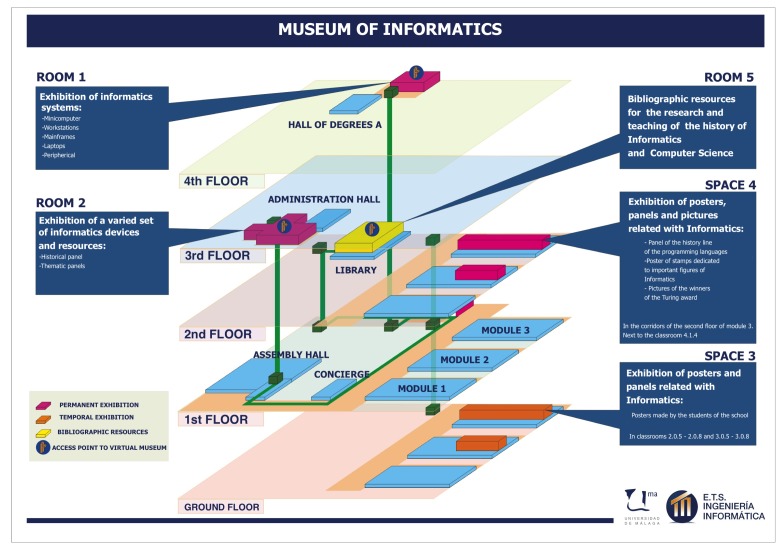
Plan of the Museum of Informatics (available at [14]).

The physical layout of our museum is unusual, principally because it does not have a dedicated building; on the contrary, the resources of the museum are located in different halls and rooms of the School of Informatics of the University of Malaga (a map is provided in Figure 1). The items in the museum are scattered over different modules of the building, which are separated by several large open areas, so it takes a long time to visit it, and there are several routes that the visitor can follow to reach an exhibition hall. This feature makes the deployment of the sensor devices that monitor presence and environmental conditions more difficult, since each museum room requires customized types of sensors. In general terms, we consider that museum items are exhibited in two different types of rooms: shared halls that are used as exhibition rooms (denoted as Type 1) and dedicated rooms for displaying museum items (namely Type 2).

**Figure 2.**
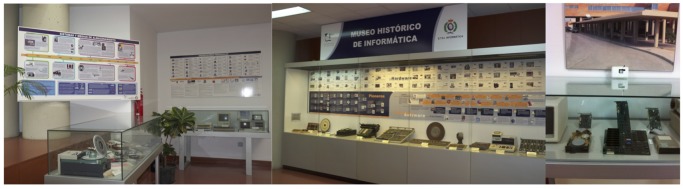
Snapshot of showcases in a Type 1 room in the Museum of Informatics.

One room where objects are on display is the administration hall. This is a public room used mainly for administration purposes. The artifacts in this hall are exhibited in seven showcases to avoid their manipulation and deterioration (see Figure 2). According to the previous classification, the administration hall of the school is a Type 1 exhibition room: a room that holds temporary exhibitions and some small museum pieces protected by the aforementioned showcases, but it is mainly seen by people just passing through on their way to administration. In this room type, sensor devices, which measure presence and environmental conditions, are by the showcases (see the snapshot to the right of Figure 2).

**Figure 3.**
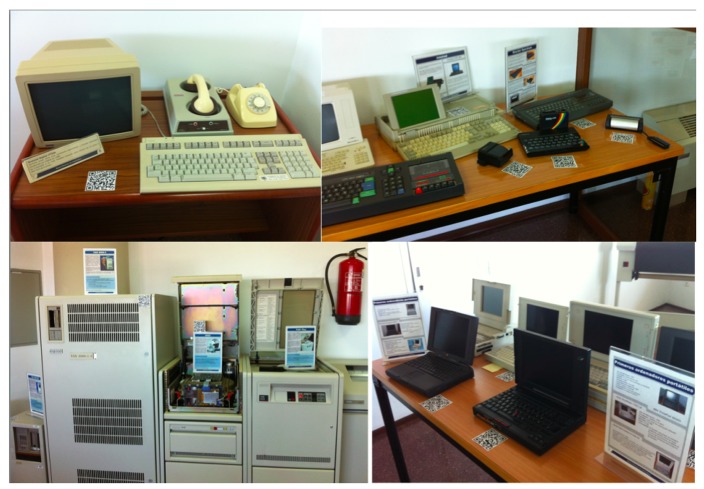
Snapshots of Type 2 room in the Museum of Informatics.

There is a dedicated room (in a module of a different building) that has different mainframes, computers and game consoles on display (see Figure 3). This room, located on the fourth floor of the library building, is a Type 2 exhibition room: it is only open to the museum's visitors. In this room, the artifacts are not safeguarded in showcases, so sensing devices are spread out all over the room. Even the presence sensing technology in the room differs from that of room Type 1.

There are several panels and posters, spread over different halls of the school, that show different aspects of informatics (such as a history line of the programming languages or stamps dedicated to important figures and events related to informatics). The corridors where the panels and posters are displayed fall into the first category, but the importance of these panels is purely documentary, so the use of sensing devices is not essential. These panels and posters incorporate (as do other labeled objects and showcases) Quick Response (QR) codes and Near Field Communication (NFC) tags that provide information about the different items in the exhibition. There is another room, located in the school's library, which stores an important bibliographic catalog of museum pieces (*i.e.*, books and manuals of some museum artifacts). The museum also has a website [14] that provides detailed information on the museum resources.

As we have already stated in the Introduction, the iMuseumA system provides three mobile-based solutions that take advantage of new technologies (such as wireless sensor networks, powered mobile personal devices and an enhanced wireless communication infrastructure) to provide context-aware services for visitors, guides and museum staff in an integrated way. The iMuseumA is an agent-oriented system, where some software agents are embedded in a set of sensors and lightweight devices. As other intelligent museums, our system includes a considerable number of sensors that provide very useful environmental and contextual information [8]. Besides this, the museum staff (guides, security crew and other members of the museum staff, such as maintenance personnel) usually carry personal hand-held devices (normally smart mobile phones), which constitute an important source of context data (e.g., location data) that can be used to provide specific context-aware services.

Table 1 summarizes the main features of the different devices deployed at the iMuseumA. For each device, a brief description and its connectivity is given. The last column (labeled function) refers to the system component that runs on it. The system is principally composed of a set of software agents running in Android-enabled personal devices, some routers and a web server that stores and provides different multimedia content about the resources of the museum. The museum also deploys sensor motes (Libelium) that provide environmental and presence data of the rooms and the showcases, and they also embed an agent. There are also some dedicated servers that hold the necessary infrastructure required by any multi-agent system (MAS), that is the agent platform (AP). The AP provides services for agents' communication and interaction. The MAS is implemented using our own agent-based solution, Self-StarMAS [15–17], which is a family of lightweight agent implementations customized to the device's restrictions on resources and its special capabilities (e.g., a sensor has less capacity and resources compared to a mobile personal device). The Self-StarMAS agents are deployed in an AP called Sol [18,19]. The necessary background to Self-StarMAS and Sol AP is explained in the next section.

**Figure 4.**
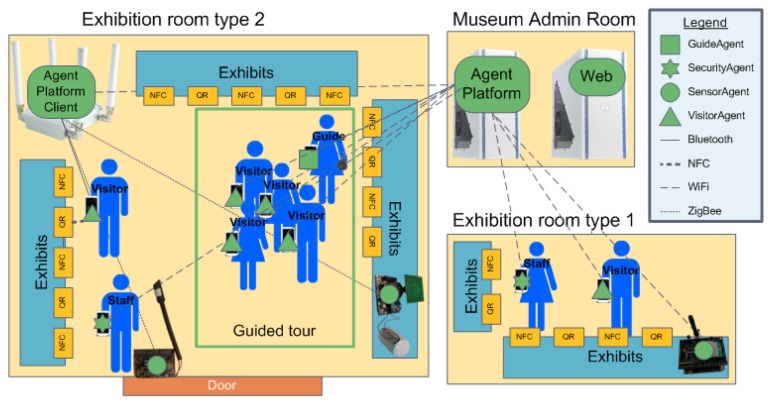
Overview of the multi-agent system (MAS) architecture of the intelligent museum.

The physical distribution of the spaces dedicated to the museum, which is included in Figure 4, determines the distributed location of the different devices in our system: (i) a couple of servers: a web server and the server where the AP is running are both located in the server room of the school; (ii) the Linux-based multi-protocol router device is located in a Type 2 exhibition room (labeled as the agent platform client (APC) in Figure 4); the different sensor motes are deployed in the different areas where objects are displayed, while the personal devices, which visitors, guides and members of the security staff usually bring with them, are present everywhere. Specifically, we have deployed different sensor motes, in the showcases of the administration hall (exhibition room Type 1, from now on, Room 1 for short) and in the dedicated exhibition room Type 2 (from now on, Room 2 for short)). The MAS, whose design and purposes will be explained in the following section, provides support to the three apps that assist visitors, guides and museum staff. The number of the other agent types is not pre-defined and depends on the number of visitors, guide and staff available at each moment.

## 3. Background: Self-StarMAS Agents and Sol Agent Platform

Currently, there exist different agent platforms, which are suitable for programming and executing AmI applications as multi-agent systems. In recent years, the demand for AmI systems has increased enormously, and many of these systems have been effectively developed with agent technology [13,20,21].

In order to ensure a more effective use of agents in developing AmI systems, general purpose agent technologies, i.e., agent architectures or toolkits and agent platforms (APs), have released new versions specifically for lightweight devices (e.g., Jade-Leap [22,23], *μ*FIPA-OS [24]) and new ones have been proposed (e.g., Andromeda [25], MAPS [26]). They provide a more or less complete framework or toolkit to facilitate the implementation of agent-based applications; and an AP principally to support the execution, registration and interaction of agents installed in lightweight devices and sensors, typical of AmI systems.

Table 2 summarizes the main features of the agent platforms (first column) that are usually used for developing multi-agent systems for AmI environments. The set of agent platforms includes both general-purpose agent toolkits for lightweight devices (such as Jade-Leap or Andromeda) and different agent platforms specific to an application domain or to a specific device (such as Agilla [27] or MASPOT [28]). A common feature of the agent platforms being considered is that agents are embedded in devices, and their behavior is customized to the available device resources (including the device's wireless connectivity).

The features included in Table 2 help to show: (i) what programming languages are used to develop and deploy agents, and the availability of an Integrated Development Environment (IDE) to support development and deployment, as well (these features are provided in the second column); (ii) in the third column, the compliance of the agent platform with FIPA (the Foundation for Intelligent Physical Agents) [29] specifications (we focus on FIPA-compliant APs, as most AmI systems are considered open systems, and FIPA promotes the use of agents in an open environment); (iii) the set of devices where the agents can be deployed (fourth column); and (iv) finally, the wireless technologies that support the communication of agents. The last row of Table 2 includes the features of the Sol agent platform and the SelfStarMAS agents, which are discussed in detail in this section.

While a discussion about the benefits and disadvantages of using these platforms for development agents of our system is beyond the scope of this work, these features allow us to observe that: there is no agent platform that supports all three kinds of devices that we find in our system (*i.e.*, PC, personal devices and sensors), except for the Sol agent platform and Self-StarMAS agents (in the last row); and, even if they were supported, the solution would not be interoperable (this is the case of Agent Factory Micro Edition (AFME) [31]). Additionally, although FIPA compliance is a feature that has been taken into account in most approaches, interoperability between all of them is not possible. More details and studies about the use of the agent technology in the context of diverse AmI environments can be found in [37–40].

Self-StarMAS agents and the Sol AP conform to a FIPA-compliant agent system, which adapts and extends standard agent technologies to facilitate the development of AmI applications. In this system, we can distinguish two parts: Self-StarMAS, a set of cooperating agents developed for lightweight devices; and Sol, which is the middleware that provides a set of (FIPA-compliant) services for those Self-StarMAS agents running in several lightweight devices (*i.e.*, the AP).

The different versions of Self-StarMAS agents are embedded in Android devices, mobile phones with a Mobile Information Device Profile (MIDP) [41], desktop computers, Sun Small Programmable Object Technology (SPOT) motes [42] and Libelium waspmotes [43]. Self-StarMAS agents can be executed on top of different APs and using different transport protocols, by simply plugging in the correct distribution aspect. For instance, by using the Jade-Leap plug-in, Self-StarMAS agents can communicate with other agents registered in this platform. However, current APs for lightweight devices are not entirely capable of managing both device and transport protocol heterogeneity and have strong limitations to ensure communication interoperability in AmI systems. The Sol AP has been created to cope with these limitations.

FIPA-based agents require a set of services from the FIPA AP that are related to the transportation of messages between agents and to the discovery of agents and services. Sol is a FIPA-compliant AP especially well suited to developing applications for AmI environments. This AP acts as an agent-based middleware that provides a set of services for the agents and behaves as a gateway to support communication heterogeneity. Specifically, the Sol AP supports:

- Registering and discovering of agents (agent management service (AMS)).
- Registering and discovering of services (directory facilitator (DF)).
- Registration and membership of groups (group management service (GMS)), which supports the identification of a set of agents that are interested in the same type of information.
- Message communication service (message transport service (MTS)), which allows the communication between agents registered in the AP by means of Agent Communication Language (ACL) messages, extended to facilitate the group-based communication.

Note that AMS, DF and MTS are all classic services provided by any AP, but the MTS was extended to support group communication in AmI environments, in conjunction with the GMS.

Therefore, the main features of this AP are the support for the communication of agents in heterogeneous devices, coping with heterogeneous transport protocols (WiFi, Bluetooth and ZigBee) and the group communication often required by pervasive systems. Additionally, Sol has remote nodes (agent platform clients), which communicate with the node in which Sol is running. The development of these clients has been necessary to implement applications distributed over wide areas. Sol clients support devices with low-range communication technology, such as mobile phones that use Bluetooth, Sun SPOTs and Libelium waspmotes. These clients can run in desktop computers and in Meshlium multi-protocol routers [44].

Sol AP implements multicast communication efficiently, which facilitates the distribution of the same information to a group of agents. Groups are defined, taking into account the communication needs of applications, and agents join and leave these groups at runtime on the fly (by the GMS). Groups are usually composed of agents that share a feature (e.g., they are embedded in the same type of device) or play the same role in the MAS (e.g., agents that provide the same service).

In summary, the combined use of Self-StarMAS and Sol provide the necessary means to develop AmI systems. Self-StarMAS agents can take advantage of using the Sol AP, so that they can communicate through different transport protocols and send multicast messages to a group of related agents. With this approach, the functionality of the AmI system is decomposed in a set of Self-StarMAS cooperating agents that use the Sol AP for location and communication between agents. Sol enables interaction via registration and discovery services.

## 4. Design of the Intelligent Museum

In this section, we outline the design of the MAS that supports our intelligent museum. As stated, the MAS presented in Section 2 is deployed in devices that comprise the museum (see Table 1). The MAS includes four different types of agents: *GuideAgent, SecurityAgent, VisitorAgent* and *SensorAgent*. Agents for mobile phones (the first three agent types) are downloaded and executed as mobile apps.

### 4.1. Design of the Agents

This subsection is devoted to showing the internal design of the agents for the sensors that make up the museum, which carry out the monitoring and control of the two exhibition rooms of the museum. The design of agents for hand-held devices (*GuideAgent, SecurityAgent* and *VisitorAgent*) has been presented in previous papers [16,38,45] and, so, is beyond of the scope of this paper. *SensorAgents* of the iMuseumA have been developed for and deployed in devices provided by Libelium [46]. Libelium provides a sensor mote called waspmote [47] that can be expanded using special modules called sensor boards. In addition, there are more than 60 sensors available for connection to the waspmote (presence, humidity, temperature, vehicle detection, luminosity, *etc.*), and there are 11 different wireless interfaces for the waspmote, including long range (3G), medium range (ZigBee, 802.15.4 and WiFi) and short range (RFID, NFC and Bluetooth 4.0).

**Figure 5.**
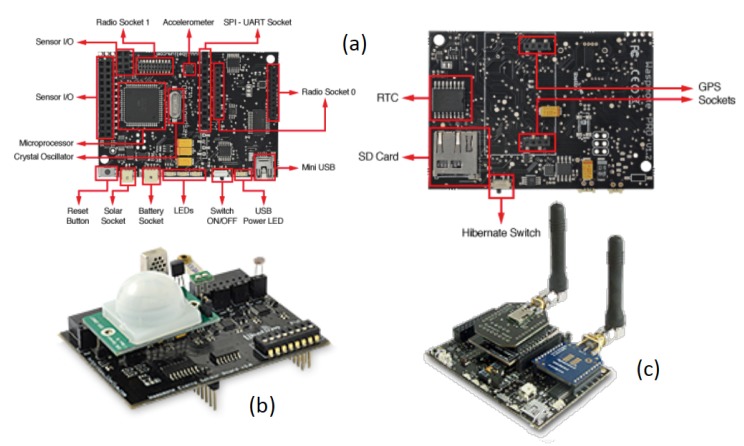
Components of a Libelium sensor device (extracted from [47]): (**a**) Top (left) and bottom (right) view of a waspmote; (**b**) events sensor board; and (**c**) expansion radio board.

Sensors are plugged into waspmotes on boards called sensor boards. Libelium provides twelve sensor boards that allow connecting to the waspmote sensors specifically for gathering different ambient and environmental data. Specifically, we have used in the museum the so-called events sensor board (in Figure 5), which allows sensors of luminosity, presence (Passive Infrared Sensor—PIR), pressure/weight, vibration, impact, Hall effect or temperature, just to mention a few to be connected. The use of the event sensor board is more than adequate for applications concerned with security (detection of vibrations, Hall effect for doors and windows, person detection with a PIR sensor), emergencies (including sensors for presence detection, water level sensors and temperature) and control of goods in logistics (using vibration and impact sensors). Regarding communication, wireless interfaces can be used alone or in combinations of pairs by using the expansion radio board (in Figure 5).

We have chosen Libelium technology, because the programming of the sensor motes is done in a language familiar to us (a variation of C) and because of the great variety of sensory and communication technologies supported. Additionally, Libelium technologies are well documented and offer support to their clients through a forum and personalized help. These features justify the selection of Libelium products for providing environmental data in our museum. More technical details are provided in [43].

**Figure 6.**
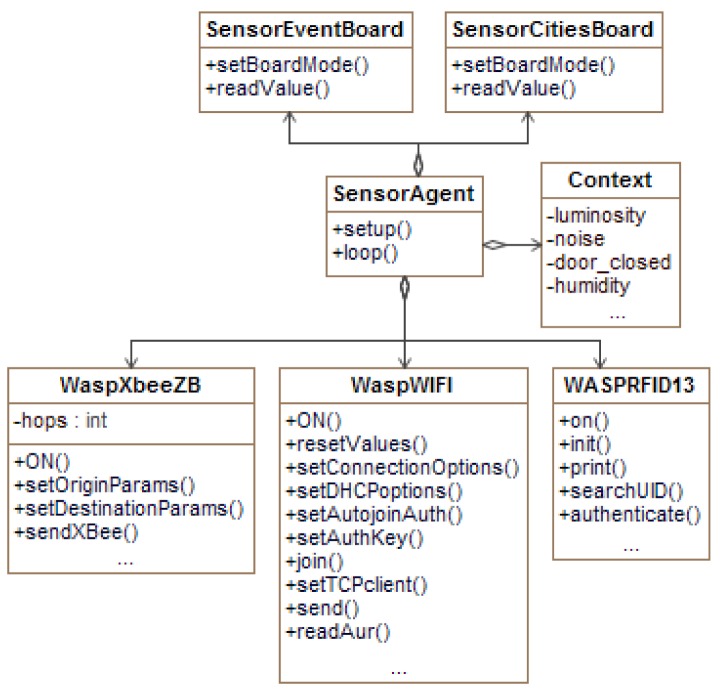
UML class diagram of *SensorAgents* running in Libelium waspmotes.

The agent design (which is presented in the Unified Modeling Language (UML) class diagram in Figure 6) for sensor motes is simple, considering the limitations imposed by the resources of sensor motes, such as battery life, memory and processing capabilities, and power consumption.

The main class, named *SensorAgent*, has two methods: one initialization method (for example, it loads all of the necessary libraries); and another method that defines the main behavior of the agent (i.e., sensing and processing environmental data). Context data (current luminosity, temperature, presence, and so on) is represented internally by the *Context class*. This class allows setting and consulting the contextual data gathered from the sensor connected in the waspmote. Functions and libraries specific to the plugged sensor board are encapsulated by the *SensorEventBoard* class for the event sensor board [48], and the *SensorCitiesBoard* class for the smart city board [49]. Libraries and functions concerning communication are encapsulated by specific classes (*WaspXbeeZB* class for ZigBee, *WaspWIFI* class for WiFi and *WaspRFID13* class for RFID). These classes deal with the creation and distribution of ACL messages to other agents through the agent platform communication services.

**Figure 7.**
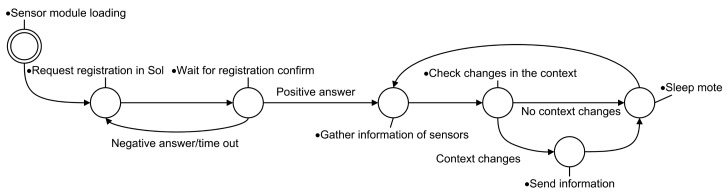
State transition diagram representing the main behavior of *SensorAgents* running Libelium waspmotes.

Figure 7 illustrates, by means of a state transition diagram, the principal behavior of the agent. In their initialization, *SensorAgents* load the library of the sensor board in which they are plugged and the libraries of the communication interfaces to which they have attached (initial state). After this initialization, the *SensorAgent* requests its registration to the platform and waits for a response. The agent retries its registration until it succeeds. Once registered, the agent starts its principal activity, which consists of gathering information from the sensors connected in the sensor board and processing these data. The new value is stored in the corresponding resource of the agent internal context. If changes in the context are detected, the agent composes a message to send this information, using the available communication technology to the corresponding agents. A sampling frequency determines when data is gathered from the sensors. In Section 6, we detail the sensors attached to the sensor boards that we have deployed in our museum.

### 4.2. Communication and Groups of Agents inside the MAS

**Figure 8.**
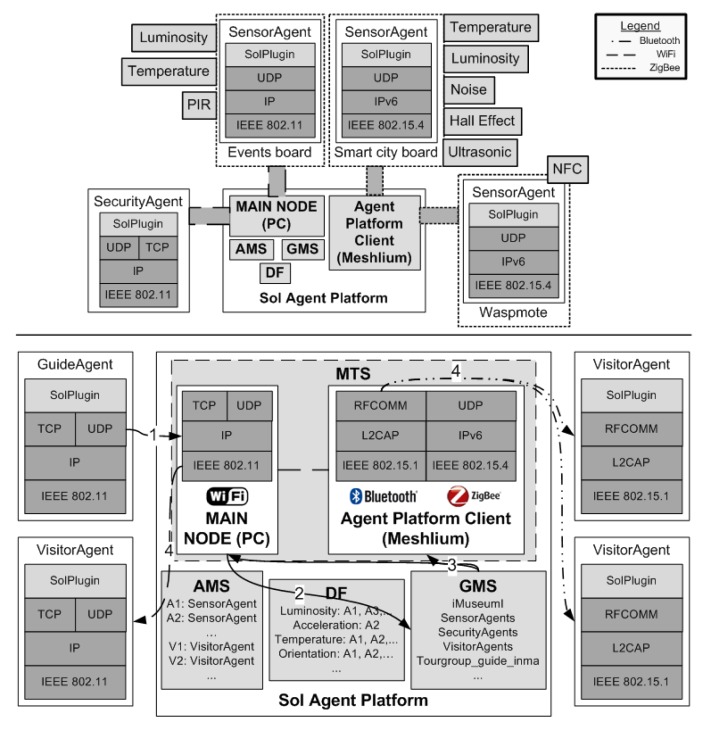
Schema of communication between heterogeneous agents in the devices of the iMuseumA (intelligent museum with agents) (**top**); and group communication inside the iMuseumA (**bottom**).

As stated, the agents of the iMuseumA communicate through the Sol AP (described in Section 3). This AP acts as a middleware that, by means of a set of services, facilitates the interoperation of agents running in devices with different connectivity. In the iMuseumA system, incompatibilities are resolved by the agent platform. The schema of communication in Figure 8 (top) shows the communication protocol stacks of the Android device where a *SecurityAgent* (on the left) is using WiFi and a Libelium *SensorAgent* (on the right) is using Zigbee. In the iMuseumA, a node of the Sol AP (running in a PC or in a Meshlium multiprotocol router) acts as a gateway between both agents. The Sol AP interconnects networks with different architectures and protocols at all levels of communication, including the agent level (which includes the formatting and transporting of agent messages).

**Figure 9.**
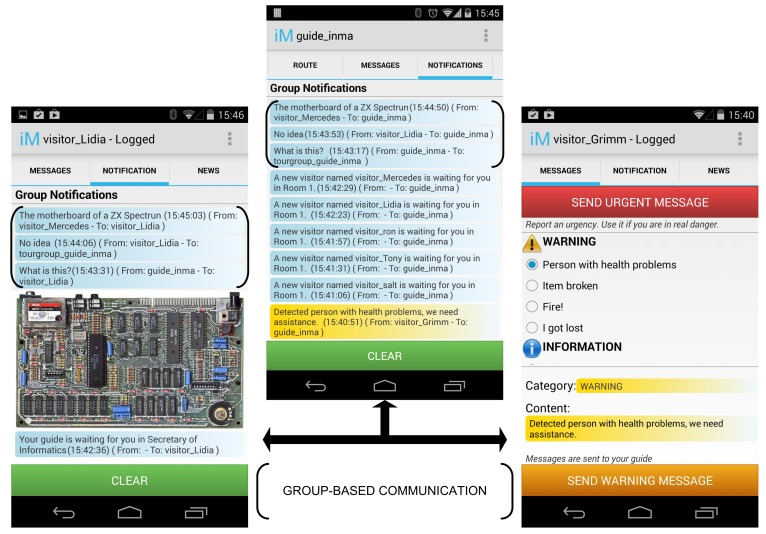
Screen shot notification tab of the guide and two visitors during a tour.

Another important aspect in agent communication is the meaning of the information that is managed and exchanged between agents. In MASs and agent-based systems, any data and information (which is known as knowledge) managed by both users and agents is defined by means of ontologies. An ontology is a representation vocabulary, often specialized to some domain or subject matter [50]. More precisely, ontologies contain the conceptualizations that the terms in the vocabulary are intended to capture, their properties and the relations between them. Agents use ontologies to structure interactions, to access services and to represent their internal knowledge about the environment. In our system, ontologies are used to characterize the information managed in the system, specifically the data that is perceived from the context (name, magnitude, value range) and the information that is exchanged. In our system, it is the sender (or origin) who decides the importance of the information that will be sent within a message. In the iMuseumA system, we can distinguish two different sources of information: users (i.e., guide, visitor and security staff) and agents (agents in sensors). On the one hand, the user explicitly sets the importance of the information that they transmit using their apps (see the right-hand screen shot in Figure 9). This information is highlighted in the application in different colors depending on the importance set by the user (see the central screen shot in Figure 9). The categories of importance of the messages are defined in the ontology. On the other hand, agents use a certain threshold value or a jitter in a value to classify or determine the importance of the data. For example, if the temperature of a room is over a certain threshold value or is drastically increasing, this is interpreted as a symptom of urgency, and the agent sends this message characterized by the ontology as urgent.

Social aspects are very important in the museum experience. People usually visit museums with groups of friends or as part of an organized excursion. Additionally, communication and collaboration of the different groups who work in the museum (guides and security staff) is fundamental to the appropriate functioning of the institution. In our system, any social aspect and other additional services are supported by the group management and communication mechanism provided by the Sol AP. Therefore, groups are a fundamental component of our iMuseumA, since they enable and enhance the experience of visitors and facilitate the work of museum staff. The agents of the iMuseumA MAS are organized in groups. There is an all-in group (named iMuseum) that includes all of the museum agents. This group, which is created when the first agent of the museum logs into Sol, is used to send and receive information considered by users to be important or interesting to the group's members (visitors, guides and security staff). Examples of pieces of information that are delivered to this group are upcoming events, such as the closure time of the museum, and emergencies.

**Figure 10.**
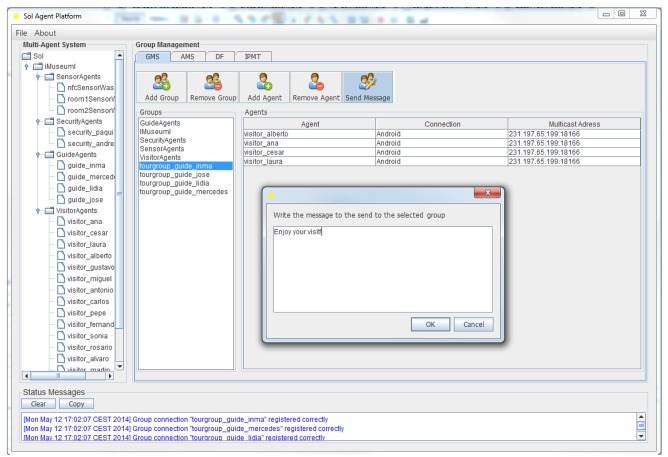
The Sol agent platform user interface for group management.

Furthermore, there is one group for each agent type that is deployed in it, so there is a group composed of all *GuideAgents*, another for *SecurityAgents, etc.* This group is used to transmit information of interest to a group or to support the coordination of the guided tours at the museum. This group membership is managed internally by each agent type once it has been created. Additionally, the museum administrator can manually include agents in specific groups using the administrator console of the Sol AP (see Figure 10).

Groups can also be created on demand and can include agents of different types. This is the case of the so-called *TourGroup* groups. This group of agents supports the communication of the members of guided tours in the museum and is composed by one *GuideAgent* and some *VisitorAgents*. Since, in the Sol AP, groups must have a unique identifier, the actual name of a tour group is the concatenation of the term *TourGroup* and the identifier of the corresponding *GuideAgent*. For example, a group of students from a school has come to visit the museum as part of a guided visit to study the history of informatics. The agent associated with this group's guide (who is a volunteer student from our department) is identified in the MAS as *guide_inma*, and the visitor agents associated with these students form a *TourGroup*, named *TourGroup_guide_inma*. The delivery of group-based communication between the members of a guided tour is illustrated at the bottom of Figure 8. The MTS of the Sol allows agents to send a message to a group of interested receivers within a group in a single transmission, even if the agents do not share the same network technology. When the guide sends a message to his/her group of visitors, the *GuideAgent* sends a unique message using the group identifier as the message target (the arrow labeled as 1 in Figure 8) through the Sol AP. This node will deal with the delivery of the message to the members of the group through the appropriate network technology. The message is forwarded to the GMS, which knows who the group members are (the arrow labeled as 2 in Figure 8). Then, the GMS arranges the delivery of the message to group members in the appropriate communication network using the MTS (the arrows labeled as 3 in Figure 8): on the one hand, for those visitor agents that support Bluetooth communication, the MTS delivers individual messages using the Bluetooth protocol stack (the arrows labeled as 4 on the right in Figure 8); on the other hand, if the target visitor agents support the TCP/IP protocol stack (associated with the WiFi network interface), the MTS sends a unique message using IP multicast (the arrows labeled as 4 on the left in Figure 8). More details about the internals of the Sol platform can be found in [19]. The creation, management, and use of these groups associated with guided tours are explained in the following section.

Apart from guides and visitors, the existence of groups is very useful for monitoring and security purposes. The existence of groups helps share information between members of the security staff and the agents running in sensing devices and enables direct communication between the museum administrator and different groups of users (to notify them of relevant information, such as the temporary opening or closing of specific rooms or notifications of broken exhibits). Additionally, this group is normally used by *SensorAgent*s to inform security staff members about people entering a Type 2 room, as explained in the section below.

## 5. Visiting the Museum

This section covers the description of the apps for visitors and guides, intended to provide information and communication to users. Most applications for intelligent museums only offer information to visitors in different languages or allow coordination between guides using radio equipment. Our system also offers this functionality, but through devices that are familiar to visitors and museum staff, that is their own personal devices.

**Figure 11.**
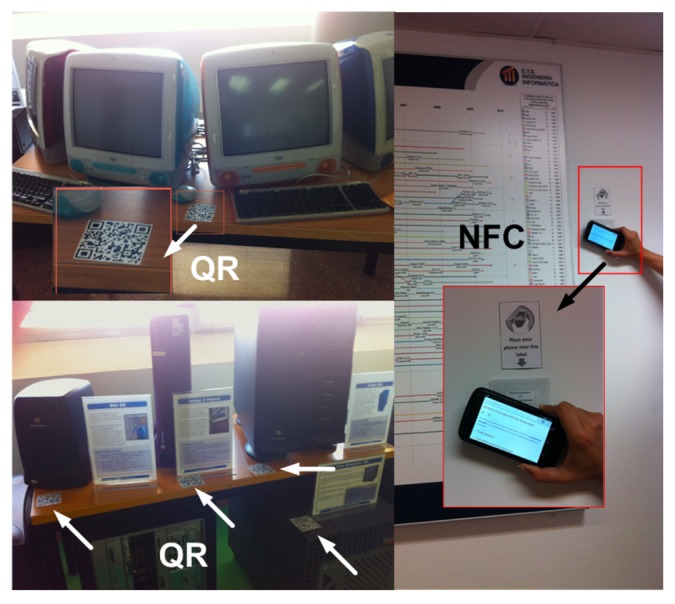
Snapshot of QR tags on exhibits. Snapshot of NFC tag on a panel located in a corridor.

**Figure 12.**
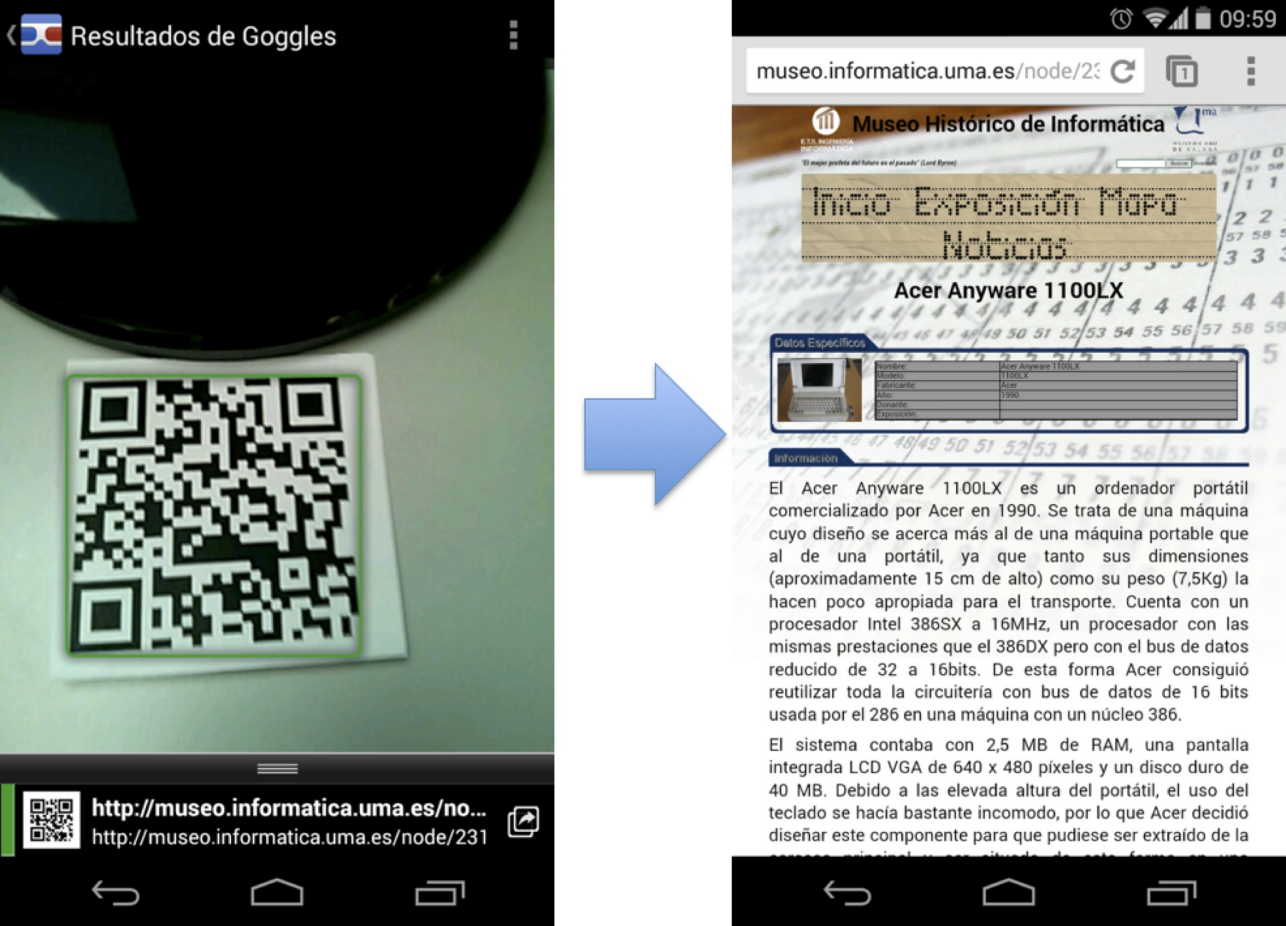
Snapshot of QR tags in exhibits and screen shot of exhibit information in smartphone browser.

In common with other museums, the application that visitors download and install permits them to gather information on the exhibits through QR code scanning (see Figures 11 and 12) or NFC tags (see Figure 11). Our museum is co-located in the informatics buildings at the University of Málaga, so its visitors are most commonly students or university staff who are curious about the exhibitions located in the shared areas of the museum. If their mobile phones are NFC-enabled, they can access the exhibition information (via web browser) just by placing their phone closer to the NFC tag (enlarged photo on the right-hand side of Figure 11). Visitors can also scan the QR code (camera in the smartphone on the left-hand side of Figure 12) and access (via web browser) detailed information about the item exhibited in front (on the right-hand side of 12).

Our system also sends alerts with news related to the museum. These notifications are sent through the Sol AP using the group of *VisitorAgents*. In addition, these visitors have the possibility to tell museum staff about any problems that they encounter during the visit. In this case, the *VisitorAgent* that represents the visitor sends messages to the *SecurityAgents* group to report any problem encountered, like broken objects, incorrect display information or any other situation that helps to improve the museum's exhibitions. As stated, the importance of this information is given by the user and characterized using the ontology of iMuseumA.

**Figure 13.**
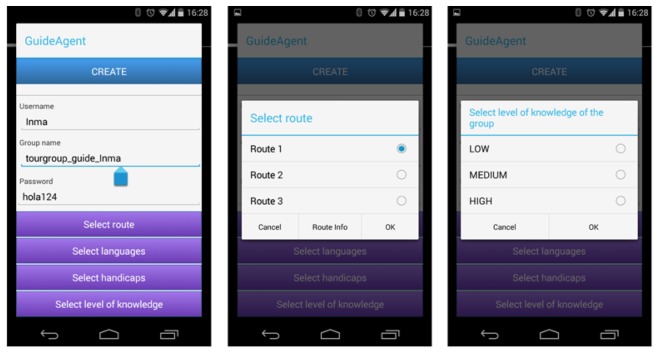
Snapshots of the guide interface for the selection of tour features.

Visitors are able to join the guided tour that better fits his/her interests and preferences (e.g., attendees of a conference held at the Informatics School). Each tour has an associated set of features (such as the spoken language). When guides organize their tours, they set up these features through their app (see Figure 13). The features considered to characterize a tour are: the planned tour inside the museum (ordered list of rooms and panels that the group is going to visit), languages used on the guided tour; disabilities of visitors that the tour is able to manage; and the level of expertise (also referred to as level of knowledge) required by the members of the group to understand the contents and explanations provided during the visit. All of these features are set by the guide according to the visitors' profiles (age, professional profile, familiarity with new technologies, *etc.*) and the type of visit (planned excursion, chance visit, *etc.*). The app allows the user to choose only one of a predefined set of options. Figure 13 shows the initial information provided by the user to create a new tour: the user name of the guide, the name used to identify the group and the password that the visitor has to supply to join the group (first snapshot on the left). After that, the guide selects one of the three planned routes (snapshot in the center of Figure 13). After setting the language to be used during the tour and the disabilities for which the tour is prepared for, the guide selects the level of knowledge of the visitors between three levels: low, medium and high (last snapshot to the right of Figure 13).

When a tour is planned, the *GuideAgent creates* a *TourGroup* and stores the features of the guided tour. When a visitor requests to join a guided tour, they indicate the disabilities they have, if any, and the level of knowledge they have about the themes of the museum (e.g., if this is their first visit, then they will probably choose a tour that requires a low level of knowledge). The languages supported are directly extracted by the agent from the user preferences of the Android system. The choice of the guided tour is performed through a negotiation.

**Figure 14.**
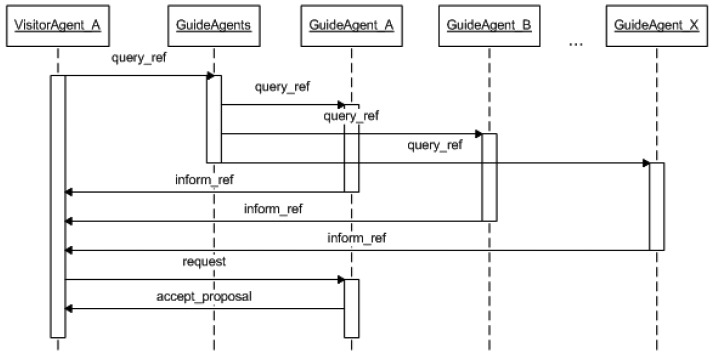
UML sequence diagram of the negotiation process between a *VisitorAgent* and a the group of *GuideAgents*.

Then, before joining a guided tour, the visitor submits a request to join a tour. The guided tour is selected after a negotiation process between the *VisitorAgent* and all of the *GuidedAgents* of the museum (see Figure 14). The negotiation starts when the *VisitorAgent* sends an ACL message with the performative *query_ref* to request information about guided tours, which is answered by the *GuidedAgents* with the features of the tours that will be starting shortly (an ACL message with the performative *inform_ref*). Once the *VisitorAgent* has received this information, it selects the tours that best fit the user's preferences and shows this information to the user. Once one of the tours has been selected, the *VisitorAgent* joins the corresponding *TourGroup* and contacts the associated *GuideAgent* (sending an ACL message with the performative *request*) to indicate that a new visitor is going to join the group and where the meeting point for that guided group will be (the starting point of the tour or the next room that will be visited if the tour has already started). Then, both the visitor and the guide receive information on the meeting point via the application. For organized tours (e.g., school excursions), the aforementioned process is unnecessary, and the visitor only needs to indicate the name of their group in the application and is then personally notified by the guide when they meet their group. When the visitor introduces the tour name, the agent automatically joins the selected *TourGroup*. Each time a new visitor joins a guided group, the guide is informed of the new incorporation of the new visitor through the notification tab (see some of these messages in the central snapshot of Figure 9). Furthermore, the visitor is notified of the meeting point where the tour will start (see the first message on the left snapshot in Figure 9).

Regardless of the process that has been followed to join a *TourGroup* (by selection of the visitor or fixed as a part of an excursion), the agent associated with the guide and all of the agents of visitors belonging to the same guided tour compose a group that is registered in the Sol AP. This group will be used during the visit by the guide to share with the visitors content about the exhibits that can enrich the experience, such as links to websites or pictures that show relevant details of the exhibits.

Group-based communication is supported by a specific service of the Sol AP, which facilitates the interaction of the member agents of the same group. During the tour, group-based messaging is a powerful mechanism that is exploited to motivate the visitors' curiosity and encourage their participation. For instance, the first and the second screen shots to the left of Figure 9 show that the guide sends an image to the visitors and encourages them to guess what the piece of hardware in the image is. The question is sent to all of the members of the guided tour, and they can either reply or ask for a clue. The responses are also shared with all of the members of the group. The messages of this interaction are shown between black brackets in Figure 9. In addition, visitors can ask the guide questions that can be answered if they are relevant to the tour. As can those visitors who are not being guided, the visitors of a guided tour can receive news related to the museum or send notifications about problems; however, these messages are directly reported to the guide of the group, who can filter them or forward them on to visitors or museum staff, if appropriate.

Guides are supported by an additional service that allows them to communicate and coordinate among themselves. This service is also supported by group-based communication and indoor positioning. The coordination between guides is performed through the exchange of messages between agents associated with the guides (*GuideAgents*) who are on a tour at that moment. The guides' app allows them to know, in real time, where all of the other guides are. To keep this information up to date with the current location, *GuideAgent*s use group-based communication. Each time a guide enters an exhibition room, the corresponding *GuideAgent* sends a message to the group of *GuideAgents* in Sol, notifying the group regarding in which room they are. Therefore, at any moment, a guide only needs to consult their device to know where other guided tours are and even to know when these are going to leave the room if the guide has given an estimation of the time needed to visit a room. This kind of service is usually not available in other museum applications.

## 6. Monitoring the Museum

Another important service of the system, which is not usually supported in an interactive way, is the monitoring and control of the different rooms of the museum. This service is particularly intended for the security staff members and is supported by the APC, *SensorAgents* and *SecurityAgents*. As stated, the monitoring of environmental and contextual information is principally performed by the sensing devices of the Libelium technology. Now, we will explain how the museum's environmental conditions are monitored and used in the two main exhibition rooms of our museum. This information is shown to the staff through the security app. Like the others in the iMuseumA app, this app enables communication with groups. This application is challenging, especially in the case of our museum, because of its physical features (objects are displayed, scattered around different rooms and halls of the school) and because of the timeshare commitment of the security personnel. In practice, museum management tasks are carried out by university staff whose primary duty is to take care of the School of Informatics' resources and support lecturers and students in teaching activities (unlocking and locking of classrooms, in charge of keys, *etc.*). Therefore, it is likely that while someone is visiting a Type 2 room, the person responsible for opening and monitoring the visit must leave their usual workstation to go to this room. Normally, at our school, rooms are locked when they are not in use, so when the visit ends, the guide must notify the porter that the room must be locked again. Our apps allow the guide and the security users to easily communicate, while the security app allows the porter to monitor and control the museum without having to be physically present in each room. It also encourages cooperation between those staff members who are responsible for monitoring the school halls (because if another member of staff is closer, they should be the one to monitor the room or open and close it if necessary).

**Figure 15.**
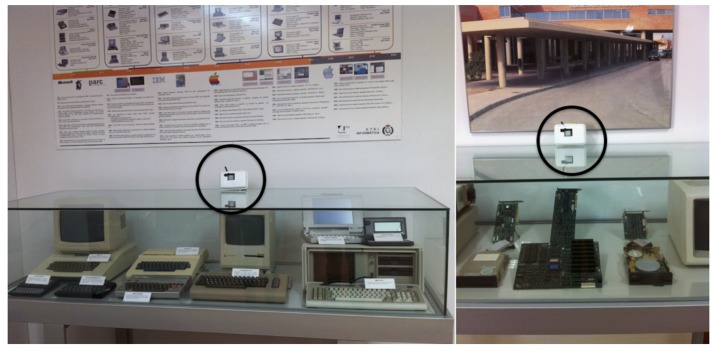
Screen shots of the showcases with the boxed waspmotes with infrared detection deployed in *Exhibition Room 1*.

As mentioned, the use of *Exhibition Room 1* is shared with the admin of the School of Informatics, so we opted for an unobtrusive mechanism with the minimum required equipment. This is a highly transited room and it arouses the curiosity of the students about the temporary exhibitions. Therefore, in this case, museum curators are specially concerned about the environmental conditions of the room and people touching the exhibits. To capture the environmental conditions, we opted to deploy the Libelium event sensor board for monitoring the showcases, because it includes sensors for measuring luminosity, temperature and presence by means of PIR sensors (see Figure 15). Regarding communications, this mote is too far away from the Meshlium router to use Zigbee-based communications (to send sensed data and interact with other agents using Zigbee radiograms), so this mote was configured with a WiFi interface (called the WiFi board) that allows the embedded *SensorAgent* to communicate with the Sol AP using TCP sockets.

The inclusion of the PIR sensor in the mote is of particular interest. The PIR sensor is a pyroelectric sensor that consists of an infra-red receiver and focusing lens whose operation is based on the monitoring of variations in the levels of detected infrared, reflecting this movement by setting its output signal high. The maximum presence detection direction is perpendicular to the sensor mote, so it is advisable to place this perpendicular to the ground (see the circles in Figure 15).

**Figure 16.**
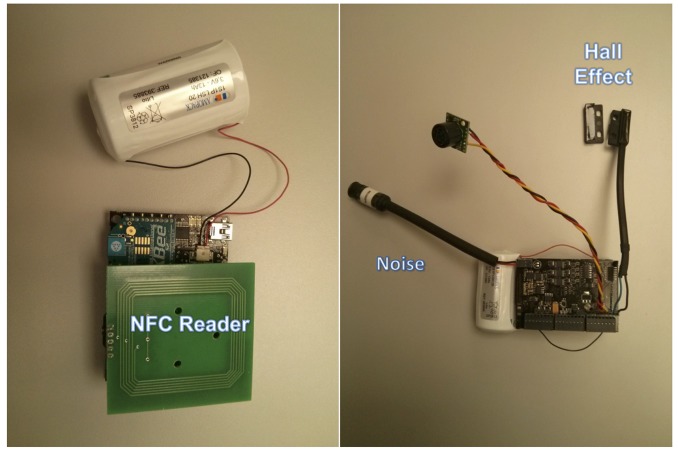
Waspmote with the expansion for the NFC/RFID reader and ZigBee (**left**) and the waspmote with the Hall effect (**right**), both used in *Exhibition Room 2*.

In *Exhibition Room 2*, the environmental conditions and the presence of people in the room are also monitored, but it only contains the Museum of Informatics. Taking this into consideration, we also deployed a waspmote and a smart city board. The waspmote has an NFC/RFID card reader to indicate the presence of different groups of visitors in this hall (see Figure 16). When a guide enters this exhibition room, he/she uses an NFC card or key chain with the NFC/RFID reader. This presence is detected by the *SensorAgent* deployed in this sensor mote, which informs *SecurityAgents* deployed in Sol that a guide with a group of visitors has entered the room.

The agent deployed in the waspmote with the smart city board performs the monitoring of the environmental conditions of the room, the volume of people in the hall and also controls whether the door of the room is closed or open. The environmental conditions are monitored using sensors for temperature, noise and light. The volume of people is measured with an ultrasonic sensor and the control of the door with a Hall effect sensor. The ultrasonic sensor attached to this node outputs an analog voltage proportional to the distance to the detected object. With this voltage value, it is possible to determine the volume of people in the hall. On the other hand, the Hall effect sensor is a magnetic sensor based on the Hall effect, which comes in two parts: one is directly connected to the sensor mote, and the other (a magnet) is attached to the door of the room. The sensor's switch remains closed in the presence of a magnetic field (when the door is closed), opening the switch in its absence (when the door is open) (see the right-hand side of Figure 16).

As *Exhibition Room 2* is dedicated exclusively to the museum, we were able to deploy low powered communication infrastructures for the sensors. Specifically, both the waspmote and the smart city board are equipped with ZigBee communication modules. The communication between the agents embedded in these sensor motes and the other agents is enabled by the multi-protocol router named Meshlium (also Libelium technology). Meshlium acts as a gateway between *SensorAgents* deployed in sensor motes and the Sol AP through the work of the APC that runs in it (see Section 4). The network technology used for the communication between Meshlium and the sensor motes is ZigBee, while Meshlium establishes the communication between APC and Sol using WiFi (the Meshlium also provides a wired Ethernet connector). Additionally, Meshlium routers offer connectivity using Bluetooth, which allows the other types of agents in the museum (*GuideAgent, SecurityAgent* and *VisitorAgent*) that are near the Meshlium to communicate with Sol using Bluetooth as the networking technology [38].

The internal behavior of these sensor devices is similar: the embedded agent reads, with certain frequency, sensed data (luminosity, temperature and presence), processes and analyses it and then delivers the information through the message transport service of the AP, to the appropriate agent. Sensed data is also used by the agent to perform simple self-management activities. For instance, when the luminosity levels determine that the room is dark (*i.e.*, nobody is in it), the sensor motes reduce their activities (sense data, send messages or even hibernate) in order to save energy and optimize resources. The reader can find more details about self-management in [15,17,51].

**Figure 17.**
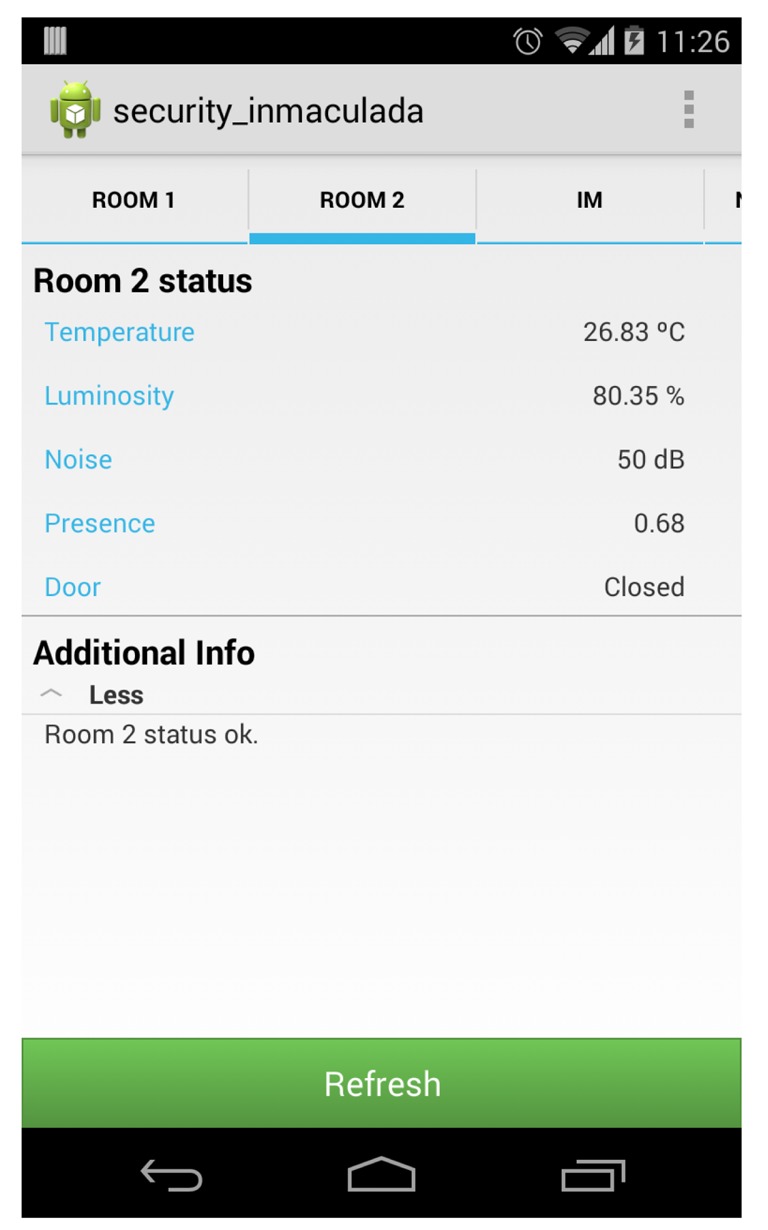
Screen shot of the monitoring info of a Type 2 room in our museum, which summarizes the data provided by sensing devices deployed in *Exhibit Room 2*.

*SecurityAgents* deployed in the security staff members' mobile phones interact with sensor agents gathering environmental information from the rooms. The app for the security staff allows contextual information of specific rooms in the museum, to be visualized (see an example in Figure 17). When the staff member in charge of guarding (a security guard or a porter) requests the information of a specific room, the *SecurityAgent* sends a message to the *SensorAgents* deployed in the room and waits until they answer with a message of their last reading. As you can see in Figure 4, information follows different paths and uses different technologies to communicate (dotted lines for ZigBee, dashed lines for WiFi, solid lines for Bluetooth and bold dashed lines for NFC) depending on the room. However, this is transparent for agents, because it is managed internally by the Sol AP.

## 7. Validation

We have evaluated the apps of iMuseumA in terms of user acceptance. The time performance of the Sol AP in terms of communication latency has been already evaluated in previous papers [17,18]. Focusing on the usability of the iMuseumA system, we conducted a user study to evaluate the user acceptance of our system. A total of thirteen participants (students and staff) from the University of Málaga were invited to visit the museum and use the corresponding app. Two volunteer students participated as guides of the museum using the app of the *GuideAgent*; three members of the University staff used the app that runs the *SecurityAgent*; and eight students used the app corresponding to the *VisitorAgent*. After the visit, they completed a questionnaire. This questionnaire, which was applied to all users, was in four parts and included an extra question for visitors and guides of the museum.

In the first part of the questionnaire, typical questions about the user profile were asked, like age, gender and familiarity with mobile applications; and also, there was a specific question that asked whether the user would recommend the application to a friend or not. The users were aged between 19 and 50 years old, and most of them were men. They use applications in mobile phones and tablets daily; only one of the users did not have an smartphone, but all of them said they would recommend the application to a friend. Additionally, in the case of guides and visitors, they were asked if the service was useful or not for finding tours. All of the users answered in the affirmative.

The second part of the questionnaire focused on general questions about the user experience; these questions and their results are summarized in Table 3. Questions were scored between one and six (six, strongly agree, and one, strongly disagree). Most of the questions were scored above four, except the question “I can use it without written instructions” for the guides. This can be explained by the quantity of functionality given to this group. This problem would be alleviated if, instead of students, the role of guides were played by permanent staff.

In the third part of the questionnaire, the users had to answer, in brief, the following questions: “Did anything about the app confuse you?”; “List the most negative aspects of the app”; “List the most positive aspects of the app”; and “What features should we add to improve the app?”. With regard to the first question, both guides and visitors agreed that the presentation of the functionality could be improved, because at some points, it is not intuitive enough. Members of the security staff did not comment on this issue. In the second question, the guides agreed that they needed previous training before using the application and that sometimes it did not work properly when the Internet access of the device was not good. In the case of visitors, they complained about the position of buttons and how they were notified of new messages from the group or from the museum administrator. Members of the security staff had no comments to make on this question. With regard to the third question, the guides were really satisfied with the functionality provided for them. The visitors appreciated the design of the interface, the usefulness of the application and the service for sharing pictures. Security staff members appreciated the facilities for communication with other members of their group and the design of the interface, and they stressed that the application is intuitive. With regard to the fourth question, the guides did not add functionality to the application and thought that a simple reworking of the application to make the interface more intuitive and fix problems with network connections was needed. The visitors' answers were really interesting, as they made some interesting suggestions about extending the functionality of the application with, for example, audio messages, outdoor location for tours that are taking place in cities, notifications using vibration, counters for unread messages, just to mention a few. Members of the staff made similar suggestions: they requested a better mechanism for notifications and audio messages.

Finally, in the last part of the test, the users were requested to evaluate their overall experience. To do this, we provided four pairs of words (terrible-wonderful, difficult-easy, frustrating-satisfying and dull-stimulating), and we asked them to choose a number between one and three, one meaning that your experience is like the first word of the pair (e.g., terrible) and three describing your experience as the second word of the pair (e.g., wonderful). Results of this evaluation appear in Table 4, and they are positive for each pair.

In conclusion, the evaluation of the application was positive for all users, especially for visitors and members of staff. The evaluation also highlighted that the app for guides, which is the most complex, must be reworked to make its functionality more evident to users. Additionally, we are satisfied by the fact that the apps provide enough functionality at this stage of the development and that the visitors enjoyed their experience with the application.

## 8. Related Work

Since the invention of the audio tour, the number and types of mobile devices used by museums have evolved a lot [1]. The increasing potential of mobile guides in museums has been underlined in several publications [2,52–55]. Nowadays, their functionalities cover a wide range of services aiming at helping users to create meaningful connections with both physical and digital environments. In terms of personal mobile devices, the majority of museums still use made-for-museums devices that are limited to voice and text messaging. Over the past ten years, almost all museums have created audio guides or provided some downloadable media content. These forms of mobile media (the traditional audio guide device, the podcast and similar downloadable content) are typically disseminated in a broadcast delivery mode: primarily for one-way delivery of content from museum to consumer. However, nowadays, two-way communication models supported by current networked mobile devices (smartphones, tablets and WiFi-enabled media players) allow richer interaction with visitors [56].

Increasingly, museums are providing visitors with smartphone applications (commonly known as apps) to enhance the user experience. These apps are intended for tours, in substitution of the traditional audio guides devices. There are many apps that provide more information than the traditional audio tours, such as for maps, schedules and wall-card information of a place. The best museum apps go beyond, frequently updating content, and even these apps serve as a useful memory aid of a museum's collection when visitors are back at home [57].

It was not until five years ago that the first museum apps appeared. In the beginning, these apps provided hundreds of images, as well as audio commentaries to accompany each image. However, the pace of app innovation is such that an image with some audio description is now considered ordinary. Today, leading apps incorporate text, audio, video and other services, like location systems. For instance, the app of the The American Museum of Natural History [5] takes full advantage of the latest technology to provide a custom navigation system. The app (named Explorer) contains all kinds of information about the collections and exhibitions in the museum, finds the visitor's current location within the museum and allows navigation using the digital floor plan, moving to the next exhibit or anywhere else in the museum using the quickest route possible. By using the WiFi network provided by the museum, Explorer can determine a user's location. The app offers step-by-step directions with arrows and maps. The system uses WiFi triangulation, allowing it to operate indoors. An important advantage of mobile applications is that apps can be updated, to include more services, context or temporary exhibitions.

Although many museums actually incorporate different kinds of multimedia guides [3–7,58], we can find the successful incorporation of new technologies that contribute to enhancing the visitor experience in museums, as well as supporting other activities, such as monitoring the environment for conservation purposes. From the beginning of the year 2000, before smartphone applications existed, we can find in the literature several approaches that exploit the advances of mobile and pervasive computing for museum environments, endowing museums with the characteristics of an intelligent environment [2,8–10,59–65]. Some of the systems considered below are prior to the proliferation of mobile devices and represented a major technological challenge at the time. We have considered them because they incorporate some features that are not supported by current museums' apps and because they represented a major technological challenge a few years back [2,63] (the amount and quality of research focusing on the application of new technologies in museums has increased significantly, and we cannot refer to them all in this section).

In 2004, the Heinz Nixdorf Museums Forum (HNF), a public computer museum in Paderborn (Germany), used an embodied conversational agent (called Max) as a guide [59]. Max was able to connect to visitors with natural face-to-face communication, providing them with information about the museum or the exhibition, while keeping up natural small talk. Max's user interface was an avatar projected on a big screen. The agent ran on a PC (not on a portable device). We have not found any evidence of whether new versions of Max were ever released. This approach has been adopted too by the Tinker virtual guide [66], a relational agent intended to increase the engagement of museum visitors.

KORE is a multi-agent system [60] that provides a personal guide for a museum's visitor, who is equipped with a Java-enabled handheld device. KORE combines infrared technology with wireless (radio) networks and uses a distributed multi-agent architecture, able to gather and filter information, according to the user profile and characteristics of the user device. The handheld device (*i.e.*, a Java-enabled PDA or cellular phone) carried by each visitor is able to recognize the user's position in the museum (by IR beamers) and even the picture, sculpture, *etc.*, that the user is looking at; on this basis, KORE can provide the user with the information, filtered and adapted to the user profile, to better understand the peculiarities of the object. The application was designed to be used in the majority of Java-enabled portable devices, and device capabilities, such as screen size, or number of colors, are automatically detected. Location awareness is obtained by means of infrared technology; the same is used to send artwork data to devices not capable of a wireless connection. There is no information about a real deployment of the system in a physical museum.

Context-awareness based on location is also the basis of the following systems. iMuseum [8] is a context-aware intelligent system that captures information about the visitor, recognizes their location and then assists them while they are visiting the museum. Each visitor is given a PDA with two context-aware applications: iGuide and iRecommender. When a visitor is interested in a cultural relic nearby, the iGuide automatically plays the corresponding multimedia recommendation of this object on the visitor's PDA. The iRecommender can recommend related relics that a visitor might be interested in after viewing some relics (according its user profile). The relics recommended are sorted by relevancy and are suggested together with their locations. Both applications, running in the PDA, connect with a server, which stores museum information, through a WiFi network. The PDA is equipped with an RFID (radio frequency identification) reader that can detect the tags attached to the relics. The iMuseum prototype system was deployed in an exhibition room of an unspecified museum. There is no news about recent versions for tablets instead of PDAs.

iMuse Mobile Tour [10] is a mobile museum guide that utilizes UHF (ultra-high frequency) RFID passive tag technology to provide context-aware information services for the Archeological Museum of Volos (Greece). Passive UHF RFID tags are attached to museum showcases to link exhibits to multimedia information. The system was implemented on a mobile RFID terminal reader (named UHF GUN by iDtronic). It comprises predefined and self-defined tours, as well as interactive games to stimulate learning. It supports multilingual and multi-audience content for tours. A distinguishing feature of this system is that it provides a service that enables the delivery of exhibit information to the members of a group on their private mobile phones to encourage learning inside groups. The service is loaded via a web browser (without any prior software installation) and pushes exhibit information to group members' personal devices. Groups are created through the iMuse Mobile handheld device, which acts as a group controller. As soon as group members connect their smart-phones to the museum's wireless network and open the web browser, a page with all available services is automatically loaded. Then, they can subscribe to a group's services by selecting the group's name. Each group subscriber is able to personalize information presentation by changing his profile. Specifically, users can change the language and the audience type.

Museum Assistant (MusA) [9] is a general framework for the development of multimedia interactive guides for mobile devices. Its main feature is a vision-based indoor positioning system that enables the provision of several location-based services, ranging from way finding to the contextualized and personalized communication of cultural content. The MusA framework allows the rapid prototyping and development of mobile guides for museums and cultural sites. As an example, the work in [9] describes the implementation of two mobile applications (one for adults and the other for children), focusing on the Palazzo Madama-Museo Civico di Arte Antica, an ancient art museum and UNESCO-listed historic residence located in the city center of Turin (Italy).

Providing context and location-aware museum guides has also been the goal and focus of several recent approaches [61,62]. In relation to context-awareness and as a first step in recommending exhibits where a visitor may wish to spend some time, there are different approaches that use mobile apps to investigate location-based predictive user models for personalized prediction of museum visitors' viewing times at exhibits [67–70].

Because of the increasing number of visitors of museums, in recent years, the problem of environmental control and safeguarding objects on display has become more and more important, and different sensor-based solutions have been sought to complement conventional monitoring methodologies traditionally used in important museums. In recent years, several scientific projects have been aimed at developing innovative tools that could complement the standard methods for environmental monitoring in museums. Different research projects have produced a new generation of passive sensors that are capable of taking into account the overall environmental effects on real works of art. The work in [71] provides a survey of these sensors, which represent a new frontier in environmental control in museums and are required to complement, in different ways, the conventional methods of environmental monitoring. In some cases, they are able to detect effects that cannot be measured by standard instrumentation. In other circumstances, these devices are used as early warning systems, that is they are intended for preliminary screening, in order to warn of possible risk situations (e.g., inappropriate lighting conditions). Once a critical situation has been identified, further controls by means of precise measurements are nevertheless required. In any case, all of these non-conventional sensors cannot be considered as substitutes for standard instrumentation, since they are capable of providing only semi-quantitative indications.

Our work (the system and the applications described in the preceding sections) shares characteristics with some of the systems and applications for museums that have been described in this section. The visitor app provides maps, schedules and up-to-date information of our museum. These apps are installed in users' smartphones at the museum (*i.e.*, commonly visitors and guides), reducing the costs, because it is not necessary to acquire and maintain specific devices. The communication between the users, when it is supported, enhances the interaction during the visit.

A distinguishing and important difference of our work, iMuseumA, compared with others is the support provided for staff members to efficiently monitor the museum exhibits using their personal mobile device (and not a specific device, like a walkie-talkie). This in an important service integrated in our museum system, which allows a richer communication with the rest of the people in the museum (visitors and guides). Note that the staff responsible for monitoring and controlling the museum are the staff of the school with shared activities, so it makes no sense that they have to carry a specific device that would only be used at certain moments. Additionally, the solutions presented above cannot support this kind of museum, where it is not possible to have a permanent staff member controlling the location and activity of the users during their visit. Remember that one peculiarity of our museum is that exhibits are scattered over different locations. Although there are some museum systems that provide automated monitoring, none of them provide this functionality integrated with the other apps. Finally, the use and benefit of group communication for heterogeneous groups of users that can be created on demand are something not commonly supported by similar museum systems. The iMuse Mobile Tour is one example of this, but it only exploits the group's creation to improve the user's visit and does not support the security staff's and guide's work, nor does it improve the interaction between security staff, visitors and guides.

## 9. Conclusions

iMuseumA provides three mobile-based solutions that take advantage of new technologies (like wireless sensor networks, powered mobile personal devices and an enhanced wireless communication infrastructure) to provide context-aware services for visitors, guides and museum staff in an integrated way. The system combines a set of sensors, lightweight devices and agent technology and interacts with users through a mobile app, which can be installed in Android-based personal devices. As for future work, we plan to incorporate the improvements suggested by users both through the evaluation and as a result of our own experimentation in our own museum. These improvements include providing a communication service between guides and the visitors to avoid noise pollution (living museum visits with teachers and administrative activities), as the noise from a visit could hinder the performance of such activities or jobs. We also aim to provide support for more smartphones (the apps), to integrate more sensing devices and to improve the location services inside the buildings by means of new technologies (e.g. Bluetooth Low Energy (BLE) or IPv6 over Low power Wireless Personal Area Networks (6LOWPan)) and services (Google Indoor Maps service).

## Figures and Tables

**Table 1.** Features of the devices of the intelligent museum.

**Device**	**Description**	**Connectivity**	**Function**
Web server	Server that hosts the museum portal	Institutional network	Exhibition and museum information
Android smartphone	Personal devices of visitors, guides and museum staff	WiFi, Bluetooth	*SecurityAgent, GuideAgent* and *VisitorAgent*
Libelium waspmote	Sensor motes	WiFi (1), NFC (1), Zigbee (all)	*SensorAgent*
Sol AP server	Server that hosts the Sol Agent Platform	WiFi institutional network	Sol AP
Meshlium	Multi-protocol router	WiFi 2.4 GHz, WiFi 5 GHz, Bluetooth and ZigBee	Agent platform client (APC)

**Table 2.** Agent platforms for AmI environments.

**Agent Platform**	**Programming Language (IDE)**	**FIPA**	**Device**	**Wireless Connectivity**
Jade-Leap [23]	Java, Java ME , Java for Android (No)	Yes	PC, MIDP , Android	802.11
*μ*FIPA-OS [24]	Java (No)	Yes	PDA	802.11
3APL-M [30]	Java, Java ME (No)	Yes	PC, MIDP, PDA	802.11
AFME [31]	Java, Java ME (No)	Yes	MIDP, Sun SPOT	802.11, 802.15.4
Andromeda [32]	Java for Android (Yes)	Yes	Android	802.11
*μ*-Agent [33]	Java for Android (Yes)	No	Android	802.11
JaCa-Android [34]	Java for Android (No)	No	Android	802.11
Jadex [35]	Java, Java for Android (Yes)	Yes	PC, Android	802.11
Agilla [27]	NesC (No)	No	Mica2	802.15.4
ActorNet [36]	NesC (No)	No	Mica2	802.15.4
MAPS [26]	Java, Java ME (No)	No	Sun SPOT	802.15.4
MASPOT [28]	Java, Java ME (No)	No	Sun SPOT	802.15.4
Sol + SelfStarMAS	Java, Java ME, Java for Android, C (Yes)	Yes	PC, MIDP, Android, Sun SPOT, Libelium	802.11, 802.15.1, 802.15.4

**Table 3.** Results for the second part of the questionnaire about the apps.

**Question**	**Average Rating**		

	**Guide**	**Security Staff**	**Visitor**
**I found this app easy to use**	4	5	4.75
**I can use it without written instructions**	2.5	4.33	4.16
**I can navigate through the app easily**	6	5	5
**The language used in the app is easily understood**	6	5.6	5.5
**Options of the application are clear**	4	5	4.87
**The prompts for input are clear**	4.5	5.33	4.14
**The app does everything I would expect it to**	5.5	5	4.25
**The app is designed for all levels of users**	5	5	4.25
**I found the various functions in the app were well integrated**	4.5	5	5.14

**Table 4.** Results for the fourth part of the questionnaire about the apps.

**Pair**	**Average Rating**		

	**Guide**	**Security Staff**	**Visitor**
**terrible-wonderful**	2	2.33	2.62
**difficult-easy**	2	3	2.75
**frustrating-satisfying**	2.5	3	2.62
**dull-stimulating**	3	2.66	2.62
